# Sugar tags and tumorigenesis

**DOI:** 10.3389/fcell.2015.00069

**Published:** 2015-11-04

**Authors:** Usha Nagarajan, Shanmugasundaram Pakkiriswami, Agieshkumar B. Pillai

**Affiliations:** ^1^School of Chemical and Biotechnology, SASTRA UniversityThanjavur, India; ^2^Department of Biology, University of Western OntarioLondon, ON, Canada; ^3^Central Inter-Disciplinary Research Facility, Sri Balaji VidyapeethPuducherry, India

**Keywords:** HSPG, glycosyltransferases, tumorigenesis, glycosaminoglycans, *Drosophila* melanogaster

The fact that the cell surface and extracellular matrix (ECM) component heparin sulfate proteoglycans (HSPGs) bind to various growth factor molecules and distribute them to targeted cell locations is known for many years (Spring et al., 1994; Nakato et al., 1995; Baeg et al., 2001; Nybakken and Perrimon, 2002; Voigt et al., 2002; Fujise et al., 2003; Johnson et al., 2004; Steigemann et al., 2004). However only during the last few years the significance of producing functional proteoglycans that regulate the signaling activities are gaining importance (Sarrazin et al., 2011). Proteoglycans are widespread from bacteria to humans with diverse expression patterns. Structural and functional features of proteoglycans possess immense ability to either promote or inhibit tumorigenesis. Members of HSPG family (glypicans, syndecans, and perlecans) function as co-receptors for several growth-related signaling pathways such as Wg, Hh, Dpp to mediate various processes like proliferation, differentiation, morphogenesis, cell-adhesion, and cell migration (Reviewed in Lin, 2004; Yan and Lin, 2009). Studies in *Drosophila* have shown that glypicans like Dally (Belenkaya et al., 2004; Han et al., 2004; Vuilleumier et al., 2010; Ferreira and Milán, 2015) and Dally-like (Dlp) (Gallet et al., 2008; Szuperák et al., 2011) facilitate movement of signaling molecules to regulate tissue growth. Recent research updates demonstrate the novel role of HSPGs in regulating additional signaling pathways like JAK/STAT (Zhang et al., 2013); PI3K and TOR (Ferreira and Milán, 2015) and also in cross-talk between signaling pathways (Wg and Dpp) to mediate tumorigenesis and metastasis (Freire-de-Lima, 2014; Herranz et al., 2014; Häuselmann and Borsig, 2014). These reports underscore the importance of studying the role of functional proteoglycans.

Interest among researchers has increased in the last few years as a result of findings in humans that list the growing number of hereditary diseases and tumors caused by mutations of the genes encoding enzymes involved in the biosynthesis of HSPGs. For example, human patients with Simpson-Golabi-Behmel syndrome (SGBS), caused by mutations in a glypican member, GPC3, suffer from tissue overgrowth that eventually develops into neuroblastomas (Pilia et al., 1996). Similarly children aged between 10 and 15 years lacking the enzymes required for synthesis of these proteoglycans, display kidney tumors called Wilm's tumors leading to eventual death (Pilia et al., 1996; Capurro et al., 2008). Few studies have also showed that distortion in the expression levels of yet another glypican family GPC1, leads to cervical and pancreatic cancers (Kleeff et al., 1998; Chen and Lander, 2001; Filmus et al., 2008). In similar lines, updates on secreted glypican, perlecans functioning as oncogenes suggested that tumorigenesis and metastasis are initiated due to the defective and non-functional proteoglycans. Defective proteoglycans are suggested to dysregulate the cell cycle and proliferation events of the neighboring host cells, thereby allowing tumor cells to invade and spread throughout the organism (Fuster and Esko, 2005; Herranz et al., 2014). However, the mechanism by which the changes in glypican function in tumorigenesis and tumor metastasis is still not clear. It is suggested that how quickly a tumor changes its properties totally depends on the tumor composition and environment.

Tumor formation and progression involves a set of unique changes in inter- and intracellular signaling. Recent reports illustrate that transformed host cells possess highly modified and non-functional proteoglycans on their cell surface (Christianson et al., 2013). These proteoglycans have been identified to promote and mediate critical patho-physiological events during various steps of tumor progression. However, only handful of factors involved in the proteoglycans synthesis are known and many more remains to be identified. In addition to the core proteins, Heparin Sulfate (HS), and Chondroitin-Sulfate (CS) chains of glycosaminoglycans (GAGs) are shown to possess specific functions. The di/tetrasaccharides linked to the core protein enable the proteoglycans to bind various signaling molecules. In functional proteoglycans, GAG-chains bind to the signaling molecules and distribute them at appropriate places while in the nonfunctional proteoglycans, GAG-chains either cannot bind to the signaling molecules or release them effectively. Problems in signaling modulation thereby leads to developmental defects and tumorigenesis. With the given importance of proteoglycans, it is highly intriguing to understand the processes underlying GAG synthesis.

Several studies have demonstrated the importance of HS chains comprising long unbranched repeats of disaccharide units of glucosamine and uronic acid. HS biosynthesis is asystematic three step process of chain initiation, elongation, and modifications. Proteoglycans biosynthesis is initiated at the GAG attachment sites on the core protein. Following this, several glycosyltransferases and modification enzymes elongate and modify the GAG chains (Esko and Selleck, 2002). Some of the enzymes known in HS GAG elongation and modifications are sugarless (sgl), sulfateless (sfl), and few *Drosophila* EXT proteins, including Tout-velu (Ttv), Sister of ttv (Sotv), and Brother of ttv (Botv) (Lin, 2004). It has been demonstrated that signaling molecules like Wg (Han and Lin, 2005), Hh (Bornemann et al., 2004; Han et al., 2004; Takei et al., 2004), and dpp (Belenkaya et al., 2004; Bornemann et al., 2004) fail to traffic in cells which are defective for components of HS GAG synthesis (encoded by *sfl, sotv, and botv* genes). Recent study showing aberrant JAK/STAT signaling due to loss of *sfl* suggested that HS chains on glypicans are indispensable for their signaling activity (Zhang et al., 2013).

Inspite of these understanding on proteoglycans, the fundamental question of how these glypicans are synthesized has not been addressed in detail. For instance, the molecules that participate in the process of chain initiation are not studied. Physiological and pharmacological evidences have been provided in other model system like rat to demonstrate the role of chain initiation step of Chondroitin-Sulfate (CS) in cell communication and development by inhibiting in proteoglycans synthesis (Margolis et al., 1991). Direct evidences from totuvelu (ttv or EXT1 in vertebrates), enzymes involved in HS-chain elongation and modifications, that function as tumor suppressors and implicated in bone overgrowth of humans (Ahn et al., 1995; Stickens et al., 1996) indicate that HS-derived GAG levels are dramatically reduced due to non-functional proteoglycans (Toyoda et al., 2000).

Chain initiation process of proteoglycans will be affected either due to modifications in the core proteins to which the initial GAG molecules are attached or mutations in the factors that transfer di/tri-saccharides to the core proteins (Baeg and Perrimon, 2000). Therefore, generation and investigation of mutants for chain initiation factors would help to explore the role of functional proteoglycans. Enzymes involved in HS chain initiation and processing are highly tissue and developmental stage specific in their function. These specific modifications enable the HSPGs in signal reception and ligand distribution. Hence mutations in biosynthetic process of glypicans would generate non-functional HSPGs, which in turn, lead to catastrophic developmental consequences (Figure 1).

**Figure 1:**
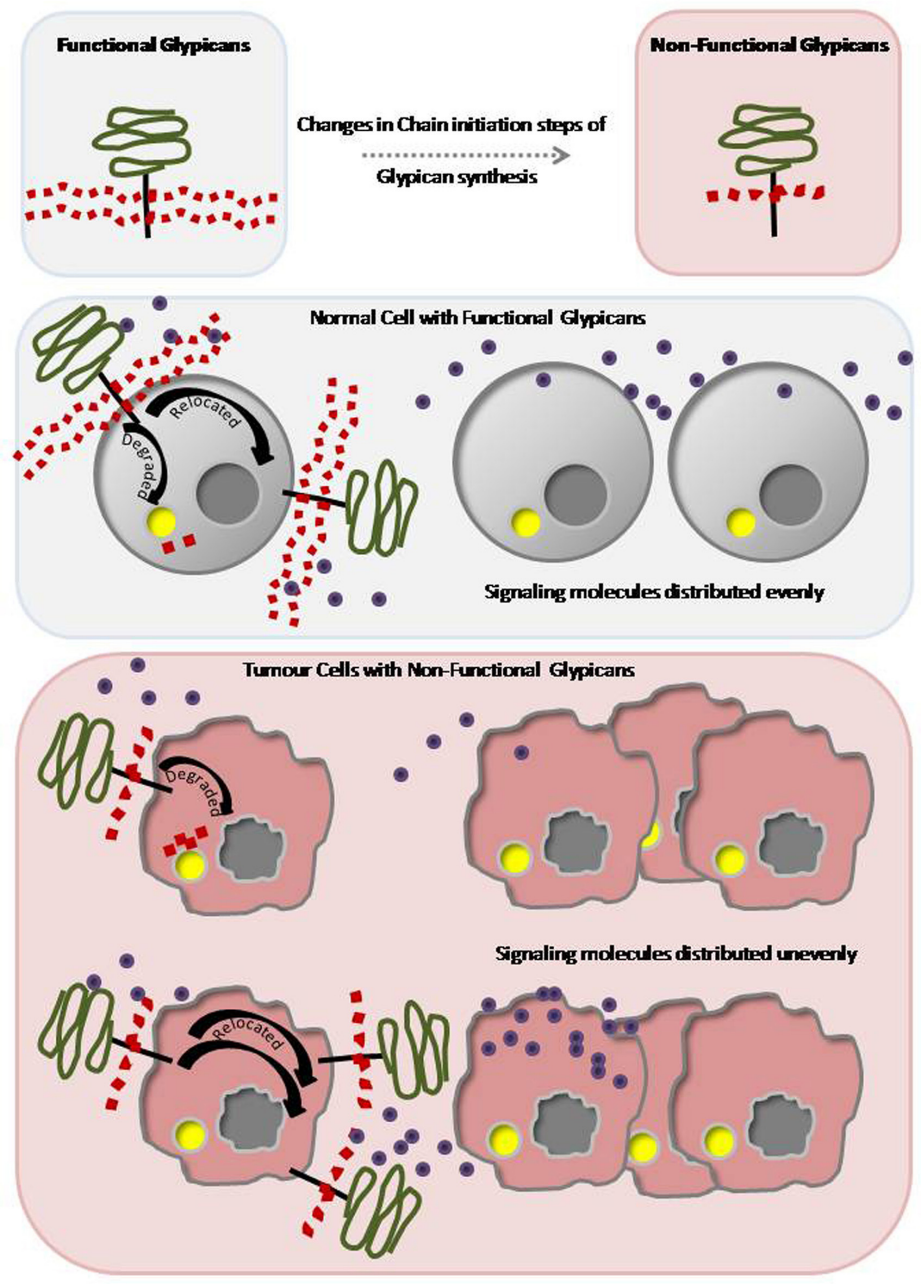
**Defective and Non-functional proteoglycans lead to developmental disorders and tumorigenesis**. Schematic representation of a functional proteoglycan with long GAG branches and a non-functional proteoglycan with short GAG branches. Any change in the chain-initiation steps of glypican synthesis leads to formation of non-functional proteoglycans. In a normal cell, functional proteoglycans with GAG branches bind to the signaling molecules. The proteoglycans are either relocated or recycled to evenly distribute the signaling molecules. In tumorous cell, defective and non-functional GAG branches cannot either bind to the signaling molecules or release them appropriately. Non-functional proteoglycans are either recycled continuously or mislocated leading to changes in distribution of signaling molecules thereby producing developmental disorders and tumorigenesis.

Till date none of the studies have characterized the role of HS GAG chain initiation-factors related to tumorigenesis. Some of the chain initiation factors like GlcAT-S, a glycosyltransferase is required for the synthesis of conserved glycosaminoglycan-protein linkage region of proteoglycans. The carbohydrate epitope Human Natural Killer 1 (HNK-1) attached by glycosyltransferase is present on several cell adhesion molecules that mediate cell-cell interactions. The HNK-1 epitope composed of specific trisaccharide (-HSO_3_-3GlcAβ1-3Galβ1-4GlcNAc-) structure is sequentially synthesized by glycouronosyl transferases (like GlcAT-S or GlcAT-P) or sulphotransferase (HNK-1ST). Glucuronyl transferase like GlcAT-S is one of the major enzymes involved in biosynthesis of proteoglycans and glycoproteins. It also modifies the Human Natural Killer 1 (HNK-1) epitope bearing ECM proteins (Pandey et al., 2011; Yamamoto-Hino et al., 2015). These chain-initiating factors are highly significant as they contribute to the rate limiting step-of proteoglycans synthesis. Therefore, any disruption to initiation process will dramatically affect the downstream reactions of chain elongation and modification generating non-functional and defective proteoglycans. Once these enzymes are identified, model organisms with defective proteoglycans can be created to address its role in maintaining tissue integrity.

To obtain a better understanding, it is now highly critical to investigate the mutant phenotypes associated with chain initiation enzymes and their interaction with core proteins. In line with this, glypican 3 (GPC-3) mutant mice show drastic developmental disorders, characterized by pre- and post-natal overgrowth. In addition, the study also illustrated Glypican 3 modulation is associated with development of endothelial, colon and ovarian cancers in adults (Filmus, 2001). Mutant animals for these glycosyltransferases tend to develop mild growth-related phenotypes like variation in organ sizes and overgrowth during the early stages of development (Filmus, 2001; Pandey et al., 2011; Yamamoto-Hino et al., 2015). During late stages of development these animals eventually display severe phenotypes due to the production and accumulation of defective and non-functional proteoglycans. Repertoire of mutants needs to be generated to precisely explore the various roles of functional proteoglycans and understand physiological conditions required for the growth factor signaling molecules binding to them at different affinities. In addition, issues related to functional redundancy among these HSPGs can be analyzed either by testing the phenotypes of double mutants or by expressing a specific HSPG core protein in the mutant background of the enzymes involved in chain initiation. Mechanisms operate in *Drosophila* to produce functional proteoglycans are similar to the one observed in vertebrates and humans. The knowledge gained in fly model may provide a further understanding into the molecular basis of adult onset diseases and tumorigenesis in humans.

## Author contributions

UN, SP, and AP designed experiments; UN performed experiments analyzed the data. UN wrote the manuscript. UN, SP, and AP revised the manuscript.

### Conflict of interest statement

The authors declare that the research was conducted in the absence of any commercial or financial relationships that could be construed as a potential conflict of interest.

## Abbreviations

ECM: extracellular matrix
HSPG: heparansulfate proteoglycan
HS: heparansulfate
GAG: glucosaminoglycans
CS: chondroitinsulfate
HNK-1: human natural killer-1
PI3K: phosphatidylinositol 3-kinase
TOR: target of rapamycin
JAK/STAT: janus kinase/signal transducer and activator of transcription.
